# Coadministration of tizanidine and ciprofloxacin: a retrospective analysis of the WHO pharmacovigilance database

**DOI:** 10.1007/s00228-020-02981-2

**Published:** 2021-01-06

**Authors:** Annette Rudolph, Hendrike Dahmke, Hugo Kupferschmidt, Andrea Burden, Stefan Weiler

**Affiliations:** 1 Pharmacology Unit, Department of Diagnostics and Public Health, University of Verona, Verona, Italy; 2 Stauffacher Pharmacy, Zurich, Switzerland; 3 Tox Info Suisse, National Poisons Information Centre, Associated Institute of the University of Zurich, CH-8032 Zurich, Switzerland; 4 Institute of Pharmaceutical Sciences, Department of Chemistry and Applied Biosciences, ETH, Zurich, Switzerland

**Keywords:** Sirdalud, Pharmacokinetics, Adverse reaction, Drug interaction, Cytochrome P450

## Abstract

**Purpose:**

Tizanidine, an alpha-adrenergic substance with antinociceptive and antihypertensive effects, is extensively metabolized via cytochrome P450 (CYP) 1A2. Therefore, coadministration with potent CYP1A2 inhibitors, such as ciprofloxacin, is contraindicated. However, both drugs are broadly utilized in various countries. Their concomitant use bears an inherent high risk for clinically significant symptoms, especially in multimorbid patients experiencing polypharmacy. This study aims to investigate the impact of coadministration of tizanidine and ciprofloxacin using real-world pharmacovigilance data and to raise awareness of this potentially underestimated safety issue.

**Methods:**

We conducted a retrospective study including Individual Case Safety Reports (ICSR) registered until March 1, 2017, in the World Health Organization (WHO) global database. Demographic data, drug administration information, the course of the adverse drug reaction (ADR), its severity, and outcomes were analyzed for cases reporting ciprofloxacin comedication.

**Results:**

In 91 (2.0%) of the identified 4192 worldwide ICSR on tizanidine, coadministration of ciprofloxacin was reported. Most of the patients were female (*n* = 59, 64.8%) with a median age of 54 years (range 13–85 years). The countries contributing most reports were the USA (*n* = 54, 59.3%) and Switzerland (*n* = 16, 17.6%). ADRs reported most often affected the nervous system and the cardiac function, especially with large tizanidine doses or drugs with CNS and cardiovascular depressant effects. In two cases, a fatal outcome was reported.

**Conclusion:**

Despite the existing formal contraindication, the concomitant use of tizanidine and ciprofloxacin can be observed in real-world clinical practice. Reactions mainly affected the central nervous and the cardiovascular system resulting in potentially severe adverse effects. The concomitant use of tizanidine and ciprofloxacin should absolutely be avoided.

## Introduction

Tizanidine is an alpha-adrenergic substance reducing spasticity [1, 2]. It also has antinociceptive and antihypertensive effects. Therefore, it is indicated for the treatment of painful muscle spasms, especially caused by multiple sclerosis, brain, or spinal cord injuries. In Europe, tizanidine is licensed in 21 EU/EEA member states. In Switzerland, it is licensed since 1983, in the UK since 2007, and in Denmark and Germany since 2009 [3].

Tizanidine has a low oral bioavailability with a high first-pass effect, considerable interindividual variability, and a narrow therapeutic range [2, 4]. It is extensively metabolized via cytochrome P450 (CYP) 1A2 in the liver before it reaches the systemic circulation [2]. Therefore, coadministration with CYP1A2 inhibitors is not recommended and a formal contraindication exists for patients treated with strong CYP1A2 inhibitors, such as the fluoroquinolone antibiotic ciprofloxacin [5].

It was shown that ciprofloxacin increases the peak plasma concentration (Cmax) and the area under the plasma concentration-time curve (AUC) of tizanidine 6–24-fold and 4–21-fold, respectively, resulting in clinical toxicity of tizanidine [5, 6]. Increased exposure in ten healthy volunteers raised the risk of severe adverse drug reactions such as excessive sedation and severe hypotension [6]. Case reports and retrospective surveys of medical records after concomitant administration of tizanidine and ciprofloxacin have previously been reported [7–9].

Based on clinical observations, we hypothesized that despite of the formal contraindication, concomitant administration could occur, due to overlapping therapeutic indication. However, no international analysis of real-world data on concomitant use and on severity of this drug-drug interaction is available. Therefore, we conducted a retrospective analysis of the World Health Organization (WHO) global database for Individual Case Safety Reports (ICSR) using a case-non-case approach to analyze this potentially underestimated drug safety issue.

## Methods

In this retrospective descriptive study, we searched for ICSR on suspected ADR in the WHO global database VigiBase^TM^. Since 1968, VigiBase^TM^, the WHO global database of ICSR, collects, processes, and homogenizes worldwide ICSR [10, 11]. These ICSR are reported by healthcare professionals and patients to the pharmacovigilance centers from more than 120 countries. VigiBase^TM^ includes mainly post-authorization unsolicited or spontaneous reports [11]. Completeness of the reports varies and is highly reporter-dependent. The ICSR data in VigiBase^TM^ include details on the demographic characteristics of the patient (age, sex), reporting country, reporter qualification (e.g., healthcare professional, patient), medications (suspect drug, interacting, and comedications), and the reported suspected drug reactions. The suspected ADRs are coded using the Medical Dictionary for Regulatory Activities (MedDRA®, latest version 23.1), and all medications are coded using WHODrug. The MedDRA® terminology follows a hierarchical structure comprising terms in five ascending orders with higher orders including more generic terms. Drug-drug interactions are included in the MedDRA® terminology as low level (LLT) and preferred terms (PT) [12]. In case of ICSR containing more than one drug, the indication of interacting drugs is possible and gets added as a MedDRA codification before being sent to VigiBase^TM^.

We included all ICSR of tizanidine reported to VigiBase^TM^ until March 1, 2017. On March 1, 2017, it contained more than 14.6 million ICSR from 126 participating countries. We received the coded data elements of all international tizanidine-associated ICSR from the WHO global database VigiBase^TM^ in the form of a Microsoft Excel file. ICSR of interest contained tizanidine (ATC: M03BX02) as a suspected or interacting drug. Combination therapy ICSR were extracted. Number of events was defined as number of unique ICSR, as identified by the report ID. All reported terms and corresponding MedDRA terms were included in the analysis as multiple ADRs can be reported in a single ICSR.

In a first step, we screened the data for ciprofloxacin as comedication. That data was then analyzed for demographic data (age at ADR onset and gender), completeness, reported ADRs, clinical outcome, concomitant drugs, seriousness, and seriousness criteria. Two subgroups were categorized by the daily dose of tizanidine with less than 12 mg and 12 mg or more per day, respectively. Other reported comedications were analyzed for CYP interactions and classified based on their effects as central nervous system or cardiovascular depressants according to the labelling in the respective summary of product characteristics (SmPC).

For descriptive analyses, we used Microsoft Office Excel (2010), IBM SPSS Statistics (version 23), and STATA (version 2017, release 15) for Windows software. Depending on the distribution of the values, we used the arithmetic mean with standard deviations or the median with ranges.

## Results

**Fig. 1:**
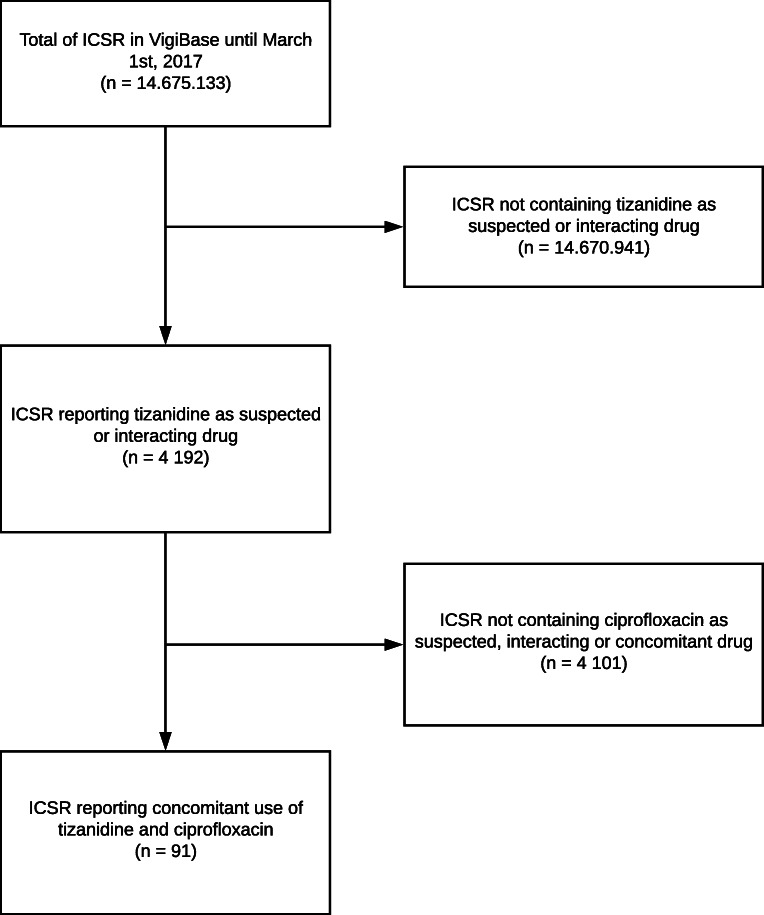
Flowchart of case selection

Worldwide, 4192 ICSR on tizanidine were identified in the WHO database in the study period. After selection of cases reporting concomitant tizanidine and ciprofloxacin administration, 91 (2.2%) international ICSR reporting in total 444 ADRs remained for analysis (Fig. 1).

**Fig. 2:**
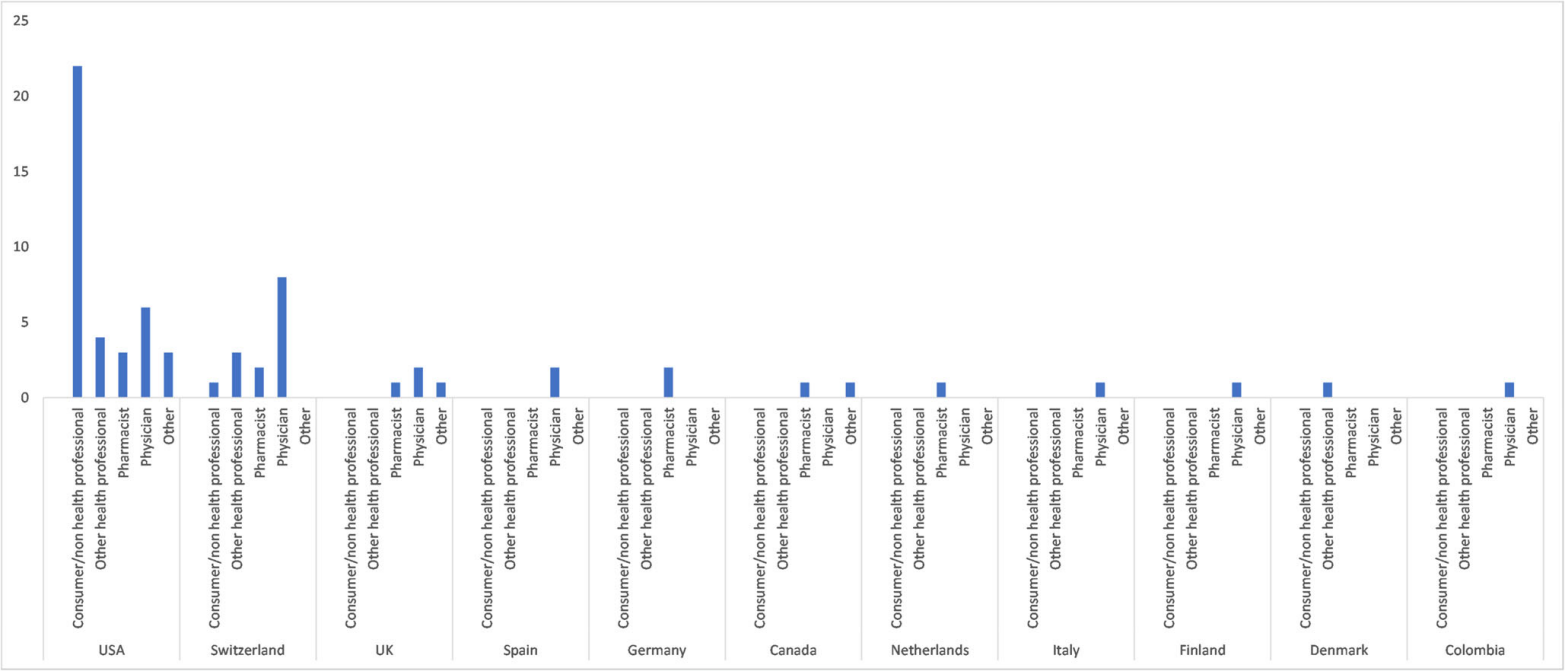
Reporting countries and primary source

In 59 cases (64.8%), the patients were female, and 28 patients (30.8%) were male (in 4 ICSR (4.4%), gender was not reported). Age was reported for 65 patients resulting in a median age of 54 years (range 13–85 years). The countries contributing most reports were the USA (*n* = 54, 59.3%) and Switzerland (*n* = 16, 17.6%). The demographic characteristics of the investigated population are summarized in Table 1. Notably, in 32 cases (35.2%), non-healthcare professionals issued the reports while physicians reported only 28 cases (30.8%) (Fig. 2). Most reports by non-healthcare professionals originated from the USA (*n* = 22, 24.2%).

**Table 1:** Demographic characteristics of investigated population

Demographic characteristics								
	Global (*n* = 91)		USA (*n* = 54)		Switzerland (*n* = 16)		Other countries (*n* = 21)	
	Total	Relative (%)	Total	Relative (%)	Total	Relative (%)	Total	Relative (%)
Sex								
Female	59	64.8	39	72.2	10	62.5	10	47.6
Male	28	30.8	12	22.2	5	31.3	11	52.4
Unknown	4	4.4	3	5.6	1	6.3		
Median age	45 (13–85 years)		51 (32–85 years)		59 (13–79 years)		55 (34–82 years)	
Serious	52	57.1	31	57.4	12	75.0	9	42.9
Seriousness criteria								
Death	2	2.2			1	6.3	1	4.8
Caused/prolonged hospitalization	24	26.4	16	29.6	5	31.3	3	14.3
Disabling	1	1.1	1	1.9				
Life threatening	8	8.8	6	11.1			2	9.5
Other medically important condition	16	17.6	8	14.8	6	37.5	3	14.3

A severity assessment was performed for most cases (*n* = 76, 83.5%). Of those, most ICSR (*n* = 52, 57.1%) were qualified as serious. The most frequent reason for severity was “caused/prolonged hospitalization” (*n* = 24, 26.4%). Two ICSR reported a fatal outcome.

Among the 444 reported ADRs, the most frequently observed reactions were “hypotension” (*n* = 25, 5.6%), “drug interaction” (*n* = 23, 5.2%), “asthenia” (*n* = 8, 1.8%), and “urinary tract infection” (*n* = 8, 1.8%). All coded MedDRA terms with a frequency of ≥ 4 are displayed in Table 2.

**Table 2:** Adverse drug reaction (ADR) characteristics for ADR being reported at least four times, categorization into nervous system disorders, cardiovascular disorders, and other ADRs. Multiple reported ADRs in single ICSR are possible

Reactions/MedDRA terms reported at least four times		
MedDRA terms	Frequency (*n*)	Number of serious ADRs (%)
Nervous system disorders		
Fatigue	7	2 (28.6)
Hallucination	7	4 (57.1)
Somnolence	7	2 (28.6)
Fall	5	2 (40.0)
Coma	4	3 (75.0)
Disorientation	4	4 (100.0)
Dizziness	4	3 (75.0)
Loss of consciousness	4	2 (50.0)
Unresponsive to stimuli	4	4 (100.0)
Cardiovascular disorders		
Hypotension	25	16 (64.0)
Bradycardia	6	5 (83.3)
Hypertension	4	2 (50.0)
Others		
Drug interaction	23	14 (60.9)
Asthenia	8	6 (75.0)
Urinary tract infection	8	4 (50.0)
Gait disturbance	6	4 (66.7)
Drug hypersensitivity	6	1 (16.7)
Labelled drug-drug interaction medication	6	3 (50.0)
Abdominal pain upper	4	2 (50.0)
Acute kidney injury	4	4 (100.0)
Decreased appetite	4	2 (50.0)
Hepatitis	4	3 (75.0)
Muscular weakness	4	3 (75.0)

The comedication was found to be very heterogenic. Among the 91 ICSR, 265 different drugs were used as comedications. The median number of administered drugs was five (Table 3). The most frequent comedication was baclofen (*n* = 18, 19.8%) followed by oxycodone (*n* = 17, 18.7%) and fampridine and gabapentin (each *n* = 15, 16.5%). Among the most frequently reported comedications, no other CYP1A2 inhibitor besides ciprofloxacin was identified. In 25 ICSR (27.5%), patients received only tizanidine and ciprofloxacin without further comedication. In 66 ICSR (72.5%), further drugs were coded. In 66 ICSR (72.5%), tizanidine was reported as suspected and in 25 ICSR (27.5%) as interacting drug. Ciprofloxacin was reported 48 times (52.7%) as suspected, 21 times (23.1%) as interacting, and 22 times (24.2%) as concomitant drug. The most frequently used nine coadministered drugs (baclofen, oxycodone, fampridine, gabapentin, hydrocodone/paracetamol, acetylsalicylic acid, pregabalin, ibuprofen, and paracetamol) were furthermore classified as antihypertensive and/or CNS depressive acting agents. Except for fampridine, all these nine drugs could cause antihypertensive ADRs, and except for paracetamol, all of them could cause CNS depressive ADRs.

**Table 3:** Top 20 reported comedications

Drug**	*n* (%)	Possible ADR*	
		CNS depression	Cardiovascular depression
Oxycodone	15 (2.6)	Yes	Yes
Baclofen	12 (2.1)	No	Yes
Gabapentin	11 (1.9)	Yes	No
Acetylsalicylic acid	8 (1.4)	No	No
Fampridine	7 (1.2)	No	Yes
Paracetamol	7 (1.2)	No	No
Magnesium	6 (1.0)	No	No
Metoprolol	6 (1.0)	Yes	Yes
Pregabalin	6 (1.0)	Yes	Yes
Alprazolam	5 (0.9)	No	Yes
Metronidazole	5 (0.9)	No	No
Paroxetine	5 (0.9)	No	Yes
Sertraline	5 (0.9)	Yes	Yes
Venlafaxine	5 (0.9)	No	Yes
Amitriptyline	4 (0.7)	No	Yes
Amoxicillin; clavulanic acid	4 (0.7)	No	No
Clonazepam	4 (0.7)	Yes	No
Esomeprazole	4 (0.7)	No	No
Hydromorphone	4 (0.7)	Yes	Yes

*Drugs were labelled with yes, when their Summary of Product Characteristics (SmPC) contained the terms “somnolence,” “bradycardia,” or “hypotension”

**Among the most frequently reported concomitant drugs, no further CYP1A2 inhibitors were identified

The daily dose of tizanidine in milligram was reported in 46 ICSR (50.5%). The median administered dose was 8 mg (range 1–48 mg). Regarding the dose-dependent character of the interaction, two subgroups with available dosage information in milligram were analyzed: 26 patients received a tizanidine dose of less than 12 mg/day and 20 patients received 12 mg or more of tizanidine daily. Patient characteristics were similar in both groups (50.0% vs 42.3% males, median age 57 years vs 55 years old). Proportions of serious ADRs were similar in both groups (55.0% vs 53.8%). In addition, the number of comedications was comparable in the subgroups (5 vs 6 concomitant drugs).

The daily dose of ciprofloxacin was reported in 37 ICSR (40.7%). The median daily dose patients were receiving was 1000 mg (500–1500 mg).

Of the identified 91 ICSR, 68 (74.7%) reported both tizanidine and ciprofloxacin as either suspected or interacting drugs. In only 23 ICSR (20.9%), the interaction between tizanidine and ciprofloxacin was coded as an ADR. When comparing the 68 ICSR to all 91 ICSR, the proportion of reporters’ qualification was found to be similar.

For two patients receiving the combination ciprofloxacin and tizanidine, a fatal outcome was reported. The first was an elderly male patient of 71 years who received tizanidine in a dosage regimen of 24 mg together with ciprofloxacin for treatment of a urinary infection. He developed muscle weakness and a decreased muscle tone, hypotension, and organ failure that eventually led to his death. The only concomitant coded drug was clonazepam—exhibiting CNS depressive effects. The second patient was a 49-year-old male with a medical history of gastrointestinal carcinoma, HIV disease, chronic viral hepatitis, and alcohol and tobacco use. He had received 2 mg tizanidine daily together with 500 mg ciprofloxacin twice daily for the treatment of a bacterial pneumonia. The number of concomitant drugs was extensive and contained morphine, zolpidem, oxazepam, clindamycin, prednisone, thiamine, levothyroxine, gabapentin, sodium picosulfate, acetylcysteine, esomeprazole, and a vitamin B supplement. Notably, morphine, zolpidem, oxazepam, and tizanidine were reported as interacting, whereas ciprofloxacin was coded as concomitant drug. The ADR that led to his death was respiratory distress.

## Discussion

The concomitant use of tizanidine with CYP1A2 inhibitors, such as ciprofloxacin, is formally contraindicated [1, 5]. Nevertheless, as hypothesized, their coadministration can be observed in real-world prescribing. The reasons for the combined use of these two drugs remain speculative. One possible explanation is the overlapping indication field: spasticity, e.g., caused by spinal cord injury or disease (such as multiple sclerosis), is frequently treated with tizanidine [13, 14]. Other chronic complications associated with spinal cord lesions comprise bladder dysfunction with urinary tract infections even leading to dangerous septicemia [13–15]. Therefore, these infections are usually treated early and fluoroquinolones, such as ciprofloxacin, an antibiotic alternative for acute cystitis and pyelonephritis, even though not in concordance with the guidelines [16–18]. The interaction between tizanidine and ciprofloxacin is the result of pharmacodynamic as well as pharmacokinetic properties of the two drugs. As shown by Momo et al. (2012), the frequency and severity of ADRs occurring under tizanidine are highly dose-dependent [8]. Their findings support a review of Henney and Runyan from 2008 [4]. It was shown that also ciprofloxacin’s ability to inhibit CYP 1A2 has a dose-dependent character. Ciprofloxacin at doses of 500 mg twice daily increased the peak concentration and AUC (0–∞) of tizanidine by 7-fold and 10-fold, respectively, resulting in hypotension, somnolence, dizziness, and reduced psychomotor performance [6]. Other effects resulting from the coadministration are significant decrease of heart rate, blood pressure, and body temperature [5, 9]. In the current study, the most frequently reported terms were related to the central nervous and the cardiovascular system, such as hypotension, somnolence, fatigue, and asthenia. Dose-dependent effects, however, could not be reaffirmed by the results of our analysis. No significant differences regarding the seriousness of the reported ADRs were observed between higher (≥ 12 mg/day) and lower doses of tizanidine. This circumstance may be due to the small sample size and the restrictive character of the available data.

Another pharmacokinetic aspect is not related to drug dosage but to patients’ physiologic properties. Tizanidine undergoes an extensive hepatic metabolism. According to the product information, the therapy of patients with decreased liver function therefore is contraindicated. The results of a retrospective survey and case report [9] and a non-randomized controlled trial [19] confirmed the relation between liver function and CYP1A2 activity. The results led to the assumption of pharmacokinetic alteration of tizanidine in patients with liver cirrhosis due to impaired activity of CYP1A2. However, in the WHO database, underlying diseases are frequently missing and laboratory information on the liver function was not available.

Drugs with similar ADR profiles administered together may cause synergistically amplified reactions and higher numbers of concomitant drugs may increase the risk to develop toxicity. Common ADRs of tizanidine include antihypertensive and CNS depressive effects [2, 5, 9, 20]. In our study, also the nine most frequently coadministered drugs are associated with antihypertensive and CNS depressive effects.

Most reports analyzed in the current study originated from the USA and Switzerland. Different from the other reporting countries, only the US and Swiss reports included reports from consumers and non-healthcare professionals. Compared with other countries, the USA has the pharmacovigilance system in which patients are the most involved already since 1969 [21]. This might be an explanation for the relatively high proportion of reports by non-healthcare professionals in the USA. It remains speculative, whether tizanidine and ciprofloxacin is more frequently coadministered in these countries, or if reporters are more aware of the investigated problem.

Not only because of the pharmacodynamic aspects of the possible ADRs but also due to the relatively narrow therapeutic range of tizanidine [4, 20], the average daily dose of tizanidine should be taken into consideration. The product information advises for an initial dose of 2 mg per day, which can be increased in steps of 2 mg over a 4-day interval. According to the product information, the ideal therapeutic effect will be reached with an average daily dose of 12 to 24 mg, which should be split on three to four equal single doses. A single dose should not exceed 12 mg and daily dose should not exceed 36 mg [1]. A special advice to minimize ADRs is increasing doses slowly [4]. Therefore, in a study in healthy volunteers, a single dose of 4 mg tizanidine was administered [6]. In the present study, maximum doses of tizanidine related to ADRs were 48 mg, which is more than the highest recommended daily dose. The length of treatment with tizanidine was documented only in few cases in the pharmacovigilance database. However, a long-term therapy could also lead to tolerance as withdrawal symptoms are reported [20]. In the subgroup analysis of a low number of patients, where dosing information was available, we did not observe major differences regarding the seriousness of the reported ADR in patients receiving higher (≥ 12 mg) vs lower daily doses of tizanidine.

As previously shown, the concomitant use of ciprofloxacin with tizanidine is frequently observed in the outpatient setting [22]. Considering the potentially severe consequences, the recognition and avoidance of the concomitant use of the two drugs is especially important.

Our study’s most important strength is the comprehensive dataset we used for the analysis. VigiBase^TM^, the WHO’s pharmacovigilance database, collects spontaneous reports on suspected ADRs of authorized drugs from countries all around the world with the aim to increase patient’s safety. Data are collected as ICSR representing mainly post-authorization unsolicited reports [23]. Therefore, this database represents the largest dataset for conducting studies and analyses of suspected ADRs.

However, the main limitation of this study is the data characteristics of the retrieved ICSR. The ICSR comprised in the register database VigiBase^TM^ contain neither narrative nor clinical or laboratory data. Therefore, this data do not allow for a formal assessment of causal drug-ADR relationship, but reports are based on the suspicion of the reporter. Since ICSR can only be extracted from the database, a verification of coding or modifications, such as recoding, is not possible. Completeness of documentation is highly dependent on individual reporter. Due to this, the ICSR are heterogeneous regarding completeness. Due to potentially missing timing and duration of use of comedication, simultaneous administration could be misclassified. Given the small number of the coded term “drug interaction,” it can be hypothesized that the contraindicated combination could have remained unrecognized as such. On the one hand, due to missing data on exact timing of administration, it cannot be ruled out that the drug use did not result in a major CYP interaction based on the timing. This might lead to an overestimation of the interaction problem of tizanidine and ciprofloxacin. On the other hand, an unrecognized interaction might lead to reporting only of the suspected drug, without reporting of comedication, leading to an underestimation of the issue. Overall, incidence rates cannot be provided solely based on pharmacovigilance data. Further observational studies are needed to clarify the prevalence of this contraindicated drug combination and potential country-specific prescribing. This might lead to a bias, which cannot be accounted for due to missing data. Another, commonly described, limitation in pharmacovigilance is the underreporting of ADRs in a spontaneous reporting system, which amounts to up to 94% [24]. Frequency of use of a certain drug, publicity, or nature of reactions can influence the volume of reports. Due to unidentified interactions, reactions can be interpreted to have non-medical etiology that can lead to a lower volume of reports.

## Conclusion

Conditions usually treated with tizanidine can be associated with accompanying symptoms resulting in ciprofloxacin treatment. Results from this analysis of real-world pharmacovigilance data show that despite the existing formal contraindication, both drugs are concomitantly prescribed in clinical practice.

Most reported adverse effects, such as hypotension, somnolence, fatigue, and asthenia, are related to the central nervous and the cardiovascular system. Concomitant use of cardio- or CNS-depressant drugs might aggravate these effects. The combination of ciprofloxacin with tizanidine should be absolutely avoided.
